# Strangulated Femoral Hernia Containing Perforated Appendicitis and Perforated Small Bowel (De Garengeot Hernia): A Case Report

**DOI:** 10.7759/cureus.62994

**Published:** 2024-06-23

**Authors:** Eithar A Alghueryafy, Abdulrahman H Albakheet

**Affiliations:** 1 Surgery, King Fahad General Hospital, Al Hofuf, SAU

**Keywords:** femoral hernia, de garengeot hernia, groin hernia, ruptured appendicitis, strangulated small bowel obstruciton

## Abstract

A femoral hernia containing the appendix within the sac is known as De Garengeot hernia. This condition is relatively rare but is important to recognize because it combines the complications of both femoral hernia and acute appendicitis, which are two distinct surgical emergencies. Clinical findings of a strangulated or incarcerated hernia may obscure signs of acute appendicitis. The presence of the inflamed appendix within the femoral hernia sac complicates the management of both conditions and requires careful surgical planning. We report a case of an 87-year-old female with De Garengeot hernia, complicated by perforated appendicitis and strangulated small bowel with perforation.

## Introduction

De Garengeot hernia is defined as a femoral hernia containing the appendix within the hernia sac. Although acute appendicitis is a common surgical emergency, its presentation within a groin hernia is rare. Pelvic appendix, due to its anatomical location, has a higher propensity to enter a femoral hernial sac [1]. Femoral hernias, including variants like De Garengeot's hernia, are more commonly seen in elderly women [2]. This condition can indeed present with symptoms such as a painful right groin lump [3]. Ultrasound and CT scans are often utilized to confirm the diagnosis, showing the inflamed appendix within the hernia sac [3]. The treatment plan must address both the appendicitis and the hernia simultaneously. We report a case of De Garengeot hernia diagnosed intraoperatively and successfully managed with open appendectomy and herniorrhaphy.

## Case presentation

An 87-year-old female with hypertension presented to the emergency department with a three-day history of lower abdominal pain, primarily in the right lower quadrant along with nausea and right groin painful swelling. She had no past surgical history. On examination, she was tachycardic (pulse rate: 105 beats/minute), hypotensive (blood pressure: 97/41 mmHg, mean arterial pressure: 56), oxygen saturation of 96% in room air, and she was afebrile (temperature: 36.6°C). Her abdomen was distended, with a tender, firm, irreducible mass in the right groin, 3x3 cm in size, just below the inguinal ligament medially, with mild skin erythema. There were no signs of peritonitis. Her laboratory results are shown in Table 1.

**Table 1:** Laboratory investigation results

Laboratory investigation	Result	Normal range
Hemoglobin	11.8 g/dl	13-17 g/dl
White blood cell count	10.8 x 10^9/l	4-10 x 10^9/l
Creatinine	98 umol/l	53-120 umol/l
Urea (BUN)	5 mmo/l	3.2-7.1 mmol/l
Aspartate aminotransferase (AST)	35 u/l	15-46 u/l
Alanine aminotransferase (ALT)	38 u/l	30-65 /l
Serum albumin	35 g/l	35-50 g/l

**Figure 1:**
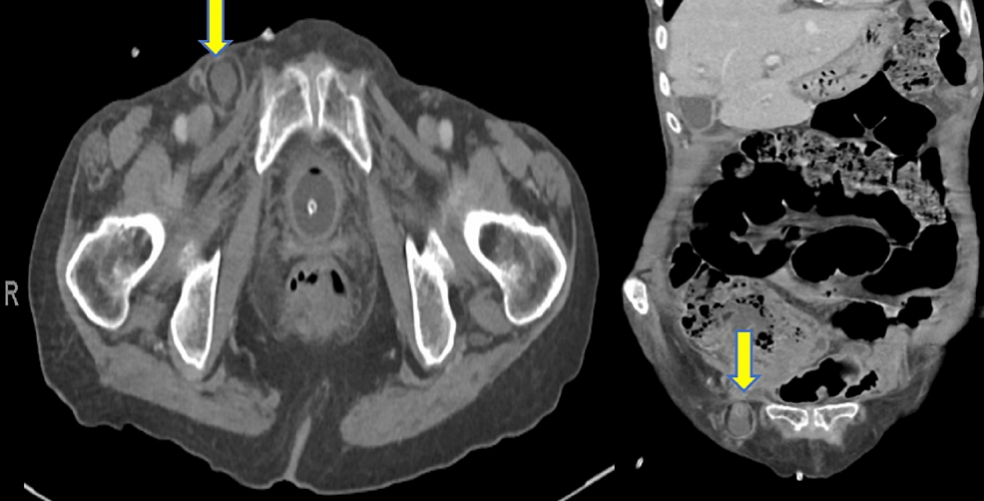
Computed tomography indicating right femoral hernial sac located inferior and lateral to pubic tubercle containing a small bowel loop with enhanced wall and free fluid (yellow arrows)

Computed tomography (CT scan) of the abdomen revealed a hernial sac measuring 3x3 cm with a narrow neck, located inferior and lateral to the pubic tubercle, suggestive of a femoral hernia. The sac contained a small bowel loop with wall enhancement and free fluid, raising suspicion of strangulation. The appendix was not visualized (Figure 1). The preliminary diagnosis was an incarcerated femoral hernia with a potentially strangulated small bowel.

**Figure 2:**
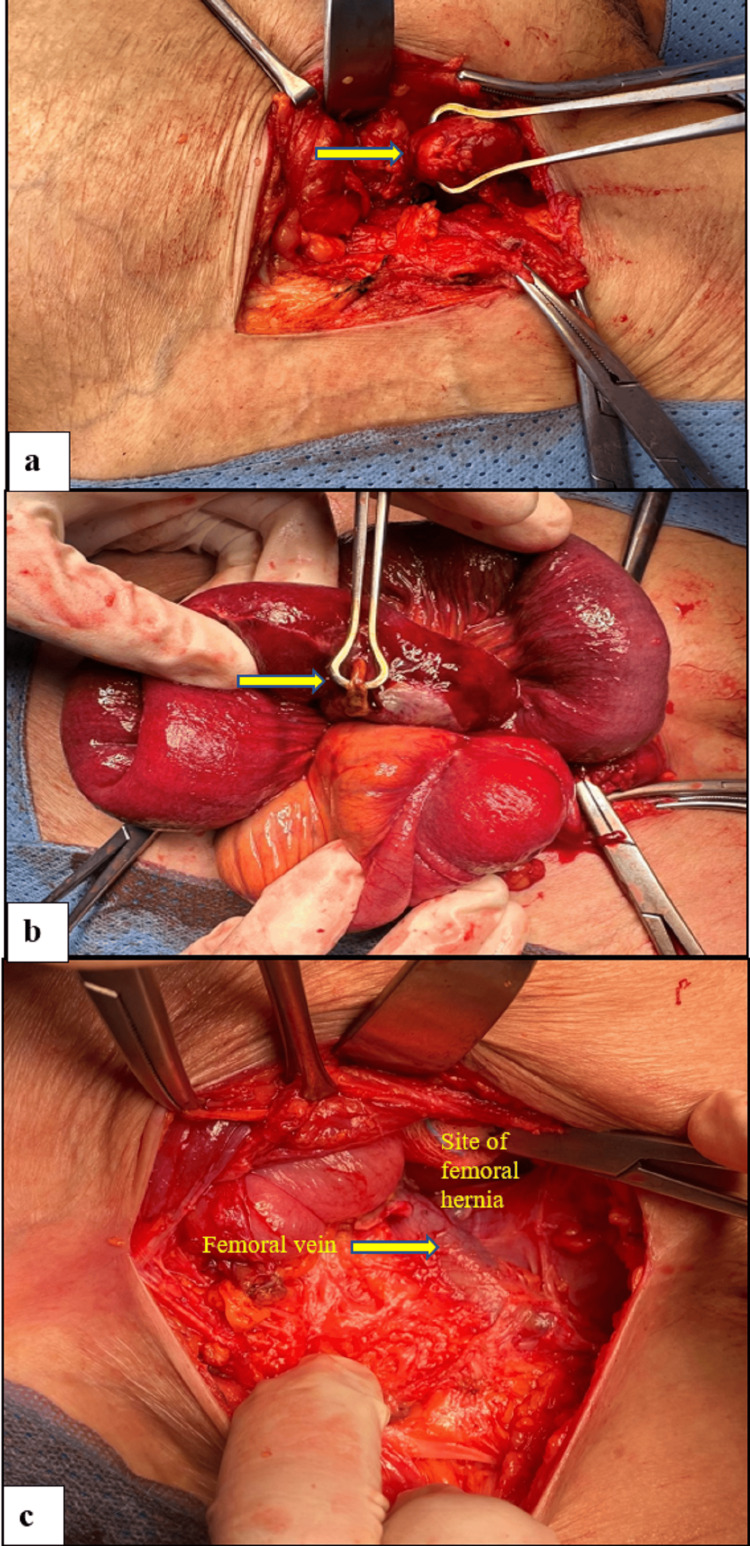
Intraoperative pictures showing a) tip of the appendix, b) perforated small bowel, and c) site of femoral hernia after reducing the contents

She was resuscitated in the emergency department with one liter of normal saline bolus, intravenous antibiotics, and analgesia, after which her vital signs improved and the patient was shifted directly to the operating room to undergo emergency open right femoral hernia repair under spinal anesthesia. Upon incision, the hernial sac was identified. The appendix tip was dilated protruding extra-peritoneally, while the base was gangrenous, perforated, and inside the sac, so appendectomy was performed. Additionally, there was a strangulated, perforated gangrenous loop of ileum inside the hernia sac, measured 15 cm, and located 110 cm from the ileocecal valve, it was resected with primary side-to-side stapled anastomosis (Figure 2). After reducing the contents, anatomical (primary) femoral hernia repair (McVay procedure) was performed by approximating the conjoined tendon to Cooper's ligament using a non-absorbable suture.

Outcome and follow-up

The surgery was uneventful. The patient was required to stay in the intermediate care unit for three days. She initially developed ileus, which resolved on the fourth postoperative day and was then transferred to the regular ward. On the seventh day, a surgical site seroma was discovered and evacuated bedside and the patient was discharged home on the same day. Histopathology results confirmed the presence of appendicular inflammation, necro- inflammatory material, and congested blood vessels. Small bowel specimens showed congestion and hemorrhage with inflamed granulation tissue. No evidence of dysplasia or malignancy in either specimen. Two weeks after being discharged, the patient visited the surgical clinic with a well-healed wound, showing no signs of infection or complications.

## Discussion

Femoral hernias protrude through the femoral canal, located medial to the femoral vein and below the inguinal ligament. They are relatively uncommon, accounting for 2-8% of all adult groin hernias [4]. Women are significantly more likely to develop femoral hernias, with a female-to-male ratio of approximately 10:1 [5]. A De Garengeot hernia occurs in only 0.5%-5% of cases when the appendix becomes trapped within a femoral hernia sac [1]. Appendicitis within a De Garengeot hernia is even rarer, with an occurrence of 0.08%-0.13% [5]. Due to its infrequent presentation, the true incidence of De Garengeot hernia is difficult to determine, but it's estimated to be between 0.15%-5% of all femoral hernias [5]. Risk factors include pregnancy-related changes, smoking, chronic cough, constipation, advanced age, and a history of inguinal hernia repair [5,6].

Amyand's hernia is described as the presence of the appendix within an inguinal hernia [5]. While incidence rates are similar, associated appendicitis is more frequently found in De Garengeot hernia [2]. Most patients with De Garengeot hernia present with a painful lump inferior to the inguinal ligament. The lump is often irreducible and tender to the touch. Erythema is seen in 33% of patients and may indicate a ruptured appendix or abscess [7]. The initial diagnosis is typically incarcerated or strangulated bowel in a femoral hernia [8]. This type of hernia carries a 15%-20% chance of strangulation due to the narrow femoral neck [5]. Sepsis and peritonitis are relatively uncommon because the anatomical structure of the femoral canal limits the spread of contaminated contents [5]. In our case, the patient did not exhibit signs of peritonitis despite the presence of appendicular and small bowel perforation. Preoperative diagnosis of De Garengeot hernia is challenging. Clinical presentation, laboratory data, and radiological investigations may be inconclusive. In most cases, De Garengeot hernia is diagnosed during surgery, with few instances where imaging leads to correct preoperative diagnosis [5]. Tsuruta et al. found a low preoperative diagnostic rate of De Garengeot hernia (17%), consistent with previous studies [2]. Plain abdominal X-rays provide non-specific findings. CT and/or ultrasonography (US) improve diagnostic accuracy, with correct preoperative diagnoses occurring in approximately 47% of patients [2]. CT scan is the most valuable tool in evaluating femoral hernia appendicitis [9]. Key CT diagnostic features include a blind-ended tubular structure located at the ventral and medial sides of the femoral vein; the tubular structure is in continuity with the cecum [2]. MRI provides comparable sensitivity and specificity to CT scan and is suitable for patients with contrast allergy [10]. Guenther et al. classified De Garengeot hernia according to the intraoperative appearance of the appendix and other involved structures within the sac (Table 2) [6]. Our findings fit the criteria of the classes 4 and 5.

**Table 2:** De Garengeot hernia classification Reference: [7]

Class	Description
Class 1	Normal appearing appendix
Class 2	2A Erythematous, inflamed, or congested appendix 2B Erythematous, inflamed, or congested appendix and erythema of the cecum or other segments of the large or small intestine
Class 3	3A Necrosis of the appendix, isolated to the tip 3B Necrosis of the appendix, involving the entire appendix
Class 4	Necrosis of the appendix and necrosis of the cecum or other segments of the large or small intestine
Class 5	Perforated appendix, abscess, or fistula

There is no standard approach to treat De Garengeot hernia, due to its rarity and limited evidence base [2]. The main goals of treatment are appendectomy, in case of acute appendicitis, and concurrent hernia repair. Several open femoral hernia repair approaches exist, including the Lockwood (infrainguinal, low incision), Lotheissen (transinguinal), and McEvedy (high) incisions. The choice depends on the patient's condition and the surgeon's preference. For emergency situations, McEvedy's high approach is the preferred one. The skin incision is made 3 cm above the pubic tubercle and extended up to the lateral border of the rectus muscle, which is divided to allow preperitoneal dissection of the hernia sac. This approach provides better access and exposure for potential bowel resection. It could also be converted to a Pfannenstiel incision if a laparotomy is needed [5]. This was the approach used in our case.

The femoral canal's narrow neck, often less than 1 cm in diameter, can make a reduction of incarcerated contents difficult without widening the canal [8]. After successful reduction and appendectomy, the preferred hernia repair method is performed. In our case, we opted for primary repair using nonabsorbable interrupted stitches to approximate the conjoined tendon and Cooper's ligament alongside the femoral vein (McVay's repair). Mesh placement remains a topic of debate [11]. Current consensus suggests that when there is no perforation or abscess, prosthetic mesh repair is feasible without increased risk of infection or recurrence [9]. However, mesh is not recommended in the presence of abscess and/or perforation [2]. In our case, we opted against mesh insertion due to field contamination from the perforated, strangulated small bowel and perforated appendix. The laparoscopic approach for De Garengeot hernia repair is controversial [5]. When imaging suggests an uninflamed appendix and a clinically stable patient, planned laparoscopic repair with mesh (such as the transabdominal preperitoneal or TAPP approach) may be recommended [2]. Comman et al. suggest that De Garengeot hernias are generally suitable for laparoscopic treatment (TAPP) [12]. The TAPP method offers the advantage of diagnostic laparoscopy, enhancing safety compared to the totally extraperitoneal (TEP) procedure [12]. The primary postoperative complications of De Garengeot hernia repair are wound infection (potentially resulting from delayed diagnosis with a prevalence of 14%-29%) and, less frequently, necrotizing fasciitis or death [5]. Our patient had an uneventful recovery period with only a small, non-infected wound seroma as a complication.

## Conclusions

De Garengeot hernia is a rare surgical condition with a challenging diagnosis. CT scans and MRIs are valuable adjuncts for diagnosing De Garengeot hernia. The choice of surgical approach for treating De Garengeot hernia can indeed be influenced by the surgeon's preference and expertise, as well as patient factors. Ultimately, the goal is to safely and effectively address both pathologies while minimizing complications. Regardless of the preoperative knowledge of the presence of the appendix within the hernia sac, the surgical management of strangulated femoral hernia must not be delayed.
